# Psychological Complications at 3 Months Following Stroke: Prevalence and Correlates Among Stroke Survivors in Lebanon

**DOI:** 10.3389/fpsyg.2021.663267

**Published:** 2021-06-10

**Authors:** Walaa Khazaal, Maram Taliani, Celina Boutros, Linda Abou-Abbas, Hassan Hosseini, Pascale Salameh, Najwane Said Sadier

**Affiliations:** ^1^Neuroscience Research Center, Faculty of Medical Sciences, Lebanese University, Hadath, Lebanon; ^2^Institut Mondor de Recherche Biomedicale (IMRB)-Inserm U955, Ecole Doctorale Science de la Vie et de la Santé, Université Paris-Est, Creteil, Paris, France; ^3^Faculty of Pharmacy, Lebanese University, Hadath, Lebanon; ^4^Institut National de Sante Publique, Epidémiologie Clinique et Toxicologie (INSPECT-LB), Beirut, Lebanon; ^5^University of Nicosia Medical school, Nicosia, Cyprus; ^6^College of Health Sciences, Abu Dhabi University, Abu Dhabi, United Arab Emirates

**Keywords:** depression, anxiety, stroke survivors, rehabilitation, stroke

## Abstract

**Introduction:** Stroke continues to be a common and debilitating medical condition which has a significant effect on public health as the second primary source of mortality and the third major root of disability worldwide. A wide range of complications affecting the survivor's life and interfering with the recovery process usually follows stroke; anxiety and depression are considered one of the major complications post-stroke. This study sought to investigate the short-term psychological consequences of stroke among Lebanese survivors and to identify their correlates.

**Methods:** This study is a prospective observational epidemiological study. 143 stroke patients admitted to hospitals in Mount Lebanon and Beirut between February and May 2018.were included in this study. Assessments of complications were carried out at 3 months post-stroke by completing a 30-min face-to-face interview questionnaire. The survey included the socio-demographic -characteristics of the patients, their lifestyle, health indicators, the severity of stroke, and the post-stroke consequences disturbing their quality of life.

**Results:** Complications were recorded for 117 stroke survivors (mean age, 72.46 years; 60.7% male). The analysis of results 3 months post stroke showed that 29 survivors suffered from neuropathic pain (24.8%), 110 (94%) suffered from fatigue, and 81 (69.2%) from cognitive impairment. High rates of anxiety (51.3%), and depression (76.1%) were recorded as well. Multivariate logistic regression confirmed that there is a significant association between depression and the following variables: anxiety (OR = 4.814, *p*-value = 0.017), pain (OR = 6.868, *p*-value = 0.002), and physical activity, which acts as a protective factor against depression (OR = 0.261; *p*-value = 0.029). However, the results of the multivariate logistic regression analysis for anxiety indicated that immobility-related complications increase the risk of anxiety by 8.457 in sedentary duration longer than 12 h (ORa = 8.457, *p*-value = 0.01). Furthermore, patients with neuropathic pain (24.8%) are 3.858 times more likely to have anxiety compared to patients without neuropathic pain (ORa = 3.858, *p*-value = 0.019).

**Conclusion:** Using a patient-centered structure more interventions should take place to evaluate stroke survivors' outcomes, and organize rehabilitation services that deal with stroke consequences, particularly high anxiety and depression levels, which are prevalent and persistent among the Lebanese stroke survivors.

## Introduction

Stroke is described as a neurological impairment due to a non-traumatic brain injury caused by cerebral infarction, or hemorrhage, which is categorized into intracerebral and subarachnoid bleeding (Easton et al., 2009). According to the “Global Burden of Diseases” (GBD), this cerebrovascular accident is rated as the second source of death and as the third most mutual root for disability worldwide (Cheah et al., 2016).

Stroke leads to various complications including musculoskeletal pain, pulmonary embolism, deep vein thrombosis, shoulder subluxation, pressure sores, spasticity, swallowing difficulties, urinary infections, and psychological problems (Kuptniratsaikul et al., 2013). According to the study of Sackley et al. (2008) that was conducted over a year after stroke, the prevalent types of complication experienced were falls (73%), contractures (60%), pain (55%), shoulder pain (52%), emotional distress (50%), and pressure sores (22%) (Sackley et al., 2008). Moreover, hemiparesis and hemiplegia are frequent and widely recognized impairments of stroke where they affect over 65% of stroke survivors (Bindawas et al., 2017). Following this cerebrovascular accident, the ability to perform daily life activities is greatly affected by physical disability, essentially upper extremities involvement, and cognitive impairment fluctuating between minor vascular cognitive impairment (VCI) and vascular dementia (VaD) (Arauz et al., 2014; Kwakkel et al., 2015). Another consequence is participation restrictions represented by loss or decline in social engagement (Scott et al., 2012). The inability to perform daily life activities and social isolation might lead to increased psychiatric complications (Bartoli et al., 2013; Hara, 2015). Diagnostic and Statistical Manual (DSM) IV defined Post-stroke depression (PSD) as a “mood disorder with the specifiers of depressive features, major depressive-like episodes, manic features or mixed features” (American Psychiatric Association, 2000; Shi et al., 2017). PSD is the most common post-stroke neuropsychiatric disorder. Depression in these patients is more prevalent than in the general population (Hackett et al., 2014; Shi et al., 2017). PSD negatively affects functional recovery, survival, cognitive and social functions. It is associated with disorders affecting attention, memory, psychomotor speed, orientation, language, and executive/motor functions (Bolla-Wilson et al., 1989; Kauhanen et al., 1999). It was demonstrated that the reported prevalence of PSD ranges between ~20 and 40% (Barker-Collo, 2007; De Wit et al., 2008; Ayerbe et al., 2011, 2013; Vansimaeys et al., 2017).

Stroke severity, deficiency of communal or household support, anxiety, and genetic factors are predisposing factors for developing depression in stroke survival patients (Ayerbe et al., 2013). Depression represents a significant load on the rehabilitation process efficacy, where it leads to more limitations in daily life activities, higher risk of intellectual deficiency, elevated chance of recurrent stroke, and greater death chance (Gillen et al., 1999; Yuan et al., 2012; Bartoli et al., 2013).

Post-stroke anxiety is another common neuropsychiatric outcome of stroke characterized by excessive anxiousness or worries, and difficulties in controlling worries. According to the Diagnostic and Statistical Manual of Mental Disorders, Fifth Edition (Kim, 2016), post-stroke anxiety is associated with the following symptoms: restlessness, insomnia, fatigue, discomfort, low concentration, and nervous tension (Castillo et al., 1995; Schultz et al., 1997; Kim, 2016). Many studies mentioned the persistence and mutuality of post-stroke anxiety (Cumming et al., 2016). Stroke survival patients are at risk of recurrent strokes, disabilities, lack of independence, and mortality, therefore, predisposing patients to develop anxiety (Johnson, 1991). Moreover, anxiety can have a detrimental effect on someone's life and lead to depression (Rafsten et al., 2018). An rate of 20% of stroke patients develop anxiety within the first 30 days following a cerebrovascular event, up to 23% in 5 months, and it reaches a value of 24% within a 6 months' length (Campbell Burton et al., 2013). Therefore, the anxiety level increases in the direction of the enduring phase of stroke (Burton et al., 2013; D'Aniello et al., 2014). Anxiety severity in this population is independent of gender. However, a negatively affected lifestyle following a stroke is linked to more severe anxiety (Rafsten et al., 2018).

Regarding the MENA (Middle East and North Africa) region, 34 studies stated the prevalence of post-stroke depression (Kaadan and Larson, 2017). The lowest rates are reported in Saudi Arabia (17%) (Hamad et al., 2011) and Iran (18% to one-third of stroke patients) (Ahangar and Hosseini, 2009; Iranmanesh, 2010), whereas higher rates are reported in Algeria (56.1%) (Layadi et al., 2013), Israel (63%) (Heruti et al., 2002), Jordan (64%) (Alghwiri, 2016), and Morocco (73.2%) (Riah et al., 2013).

Regarding Lebanon, several published studies shed the light on the prevalence, incidence, symptoms, and the needed therapeutics for stroke (Jurjus et al., 2009; El Sayed et al., 2014; Farah et al., 2015). Smoking, hypertension, diabetes mellitus, and physical inactivity were linked to a higher risk of stroke in published Lebanese studies (Lahoud et al., 2017; El-Hajj et al., 2019). Death is known as one of the major consequences of stroke, a recent study done by Abdo et al. (2019) confirmed that the cumulative mortality rates of Lebanese stroke patients increased from ~1 over 7 at 1 month to 1 over 5 at 1 year post-stroke (Abdo et al., 2019). However, no study has shed the light on the challenges faced by Lebanese surviving stroke patients.

In the light of the aforementioned literature, this study explores post-stroke neuropsychiatric complications in Lebanon and identifies their potential correlations.

## Materials and Methods

### Study Design, Settings, and Participants

An observational prospective study was performed among stroke patients survivors admitted to eight hospitals from Mount Lebanon and Beirut including Hôtel-Dieu De France, Clinique Du Levant, Mount Lebanon, Middle East Institute of Health, Al Sahel General, Al Zahraa, Rafic Al Hariri University, and Al Hayat.

Starting from February 1st, 2018, and for the subsequent 3 months, all consecutive patients admitted to the participating hospitals with a confirmed diagnosis of ischemic or hemorrhagic stroke were eligible to participate in the study. The inclusion criteria of our study considered Lebanese patients of either sex, aged over 18 years with a clinical diagnosis of first ischemic or hemorrhagic stroke confirmed by a brain CT scan or MRI and that is well-identified by the following codes based on the “International Classification of Diseases” 10 (ICD-10) (I63-I61) (“ICD-10 Version: 2016,” 2016), and classified into cerebrovascular accident, stroke, ischemic stroke, hemorrhagic stroke, intracerebral hemorrhage or embolic/thrombotic stroke. The exclusion criteria were: patient admitted for a recurrent stroke or a transient ischemic attack (TIA), patient with neuropsychiatric and/or cognitive disorders prior to the onset of the stroke such as previously diagnosed depression, anxiety, seizures, dementia, aphasia, memory and attention disorders, patients who refused to participate in the study, and patients who accepted participation at the beginning of the study and decided to stop during the first 3 months of follow-up. This article complies with the Strengthening the Reporting of Observational Studies in Epidemiology (STROBE) guidelines [44].

### Procedure

All the eligible patients were contacted via phone within the first 3 months post-stroke after collecting the baseline characteristics including socio-demographic characteristics, current health behaviors and clinical data from the medical records in the hospitals. They were informed about the purpose of the study, invited to participate in the study and to sign a consent form. After receiving the signed informed consents, a face to face interview was performed by two trained researchers in the place of residence of the patients at the 3 months after the acute event. Data gathering was performed through structured questionnaires directly from the patients or their family members and from hospital records. The questionnaire used for data collection was composed of several parts:

1. Socio-demographic characteristics: age, gender, marital status, education level, professional status, and Insurance.
2. Current health behaviors: cigarette or waterpipe smoking (yes/no) and physical activity (yes/no)
3. Clinical data: Clinical data were collected from the medical records to detect patients diagnosed with Hypertension (HTN), Atrial Fibrillation (AF), Dyslipidemia prior to stroke onset, and to specify the type and location of the stroke. Hypertension is defined as the elevation of blood pressure revealing a value equals or more than 140 mmHg for systolic blood pressure, or equals or greater than 90 mmHg for diastolic blood pressure (Chobanian et al., 2003). Atrial fibrillation (AF) is) is defined by discharge diagnosis coded from hospitals (ICD-8 codes 4279, 42793, and ICD-10 codes I48) (Gundlund et al., 2016). Dyslipidemia is defined by a history of the total level of cholesterol more than or equals 200 mg/dL or in case of the presence of lipid-lowering therapy (Matz et al., 2016).
4. A battery of tools was also administered to the patients 3 months post-stroke: Mini-Mental State Examination (MMSE) for cognitive function assessment, Hospital Anxiety and Depression Scale (HADS), Fatigue Severity Scale (FSS), “Douleur Neuropathique 4” (DN4) to evaluate neuropathic pain, National Institutes of Health Stroke Scale (NIHSS) to measure the neurological function. The required information of patients with mute; global aphasia; no usable speech or auditory comprehension were provided by their caregivers.

### Data Measurements

#### Mini-Mental State Examination(MMSE)

The MMSE was used to evaluate the cognitive impairment in research (Arevalo-Rodriguez et al., 2015). MMSE is divided into 11 criteria for the assessment of cognitive function that mainly include attention and orientation, memory, registration, recall, calculation, language and ability to draw a complex polygon. Scores fluctuate between 1 and 30 depending on each case. Lower scores designate severe cases with higher impairment. Previous research has validated the use of the Arabic version of MMSE among the Lebanese population and recommended the use of 23 as a cut off to describe “normal” intellectual function (El-Hayeck et al., 2019).

#### Hospital Anxiety and Depression Scale (HADS)

HADS is designed to measure the severity of depression and anxiety post stroke through a concise self-assessment questionnaire of 14 items. It is divided into two scales of seven components: a scale for depression and a scale for anxiety. Classically, a score of eight or more for each subscale is used as a cut-off score in studies to distinguish between cases of depression and anxiety and the unharmed. This cut-off point is also frequently used in the population of stroke and has adequate specificity and sensitivity in recognition of depression and anxiety disorders after stroke (Vansimaeys et al., 2017). The Arabic validated version of HADS was used in our study (Al Aseri et al., 2015).

#### Fatigue Severity Scale (FSS)

FSS is a self-assessed scale of nine elements to examine the severity of fatigue among the various circumstances throughout the last week. Items are answerable on a Likert scale from “1” “strong disagreement” to “7” “strong agreement.” The FSS, a measure created by adding all the items of the score, ranged from 1 to 63 with higher scores indicating greater fatigue. A value more than 4 indicates the presence of fatigue (Valko et al., 2008). The Arabic validated version of the FSS was used in our study (Al-Sobayel et al., 2016).

#### Douleur Neuropathique 4 (DN4)

DN4 is a 4-item questionnaire of Neuropathic pain designed by Bouhassira and colleagues in 2005 to diagnose central pain. It is characterized by sensitivity and specificity in recognizing chronic neuropathic pain of different etiology. DN4 includes ten elements (burning, painful cold, electric shocks, tingling, pins and needles, numbness, itching, touch hypoesthesia with a soft brush, disposable examination pins used for pinprick hypoesthesia, a soft brush used to test tactile dynamic allodynia) answerable by yes (presence) or no (absence). The total score ranges from 0 to 10. Neuropathic pain is diagnosed if the total value is 4; which is the cut-off value (Spallone et al., 2012; Bashir et al., 2017). The Arabic validated version Dn4 was used in our study (Terkawi et al., 2017).

#### National Institutes of Health Stroke Scale (NIHSS)

The outcome of stroke in the medical practice, especially the measure of the neurological function is widely evaluated through the NIHSS. It involves 11 graded criteria comprising: “level of consciousness, language function, visual fields, eye movements, facial symmetry, motor strength, sensation, coordination and so on, which has undergone extensive validation and reliable assessments” (Cao et al., 2015). This scale is marked from zero (without alteration) to a maximum of 42. Severe cases usually compromise scores of 21 or more (Harrison et al., 2013). It is grouped into four categories concerning variations in severity. “0–5 is mild,” “6–10 is moderate,” “11–15 is moderate to severe,” and “equals or more than 16 is severe impairment” (Roth et al., 2001).

### Study Size

The Epi-info 7 program was used to calculate the minimum sample size needed in the study. Considering a prevalence of stroke in Lebanon of 3.9% depending on the study of Jurjus and collaborators (Jurjus et al., 2009), assuming a confidence interval of 95% and a margin of error of 5%, the minimum sample size calculated for our study was estimated at 116 patients. To account for an estimated 10% of lost to follow-up, it is necessary to include 128 patients.

### Statistical Methods

The data entry and analyses were performed using the “Statistical Package for Social Sciences (SPSS) software version 22 (SPSS^TM^ Inc., Chicago, IL USA).” Means with their standard deviations and percentages were used to describe continuous and categorical variables, respectively. Statistical bivariate analysis was performed. The Pearson chi-square (χ^2^) test was used for categorical variables.

Multivariable analyses using logistic regression were carried out to identify the correlates of post-stroke depression and post-stroke anxiety, 3 months after stroke. Dependent variables were anxiety and depression. Independent variables for Anxiety were gender, educational level, physical activity, sedentary duration, hypertension, atrial fibrillation, dyslipidemia, neuropathic pain (Dn4), National Institutes of Health Stroke Scale (NHISS). Independent variables for Depression were gender, physical activity, educational level, employment status, cognitive impairment (MMSE), anxiety (HADS), neuropathic pain (Dn4), Fatigue (FSS), National Institutes of Health Stroke Scale (NHISS). The independent variables introduced in multivariable analyses were those showing a *p*-value <0.2 in bivariate analyses. The final logistic regression model was reached after ensuring the adequacy of our data using the Hosmer and Lemeshow test. Adjusted odds ratios and their 95% confidence intervals were reported. In all cases, a *P*-value <0.05 is considered significant.

## Results

### Participants

**Figure 1:**
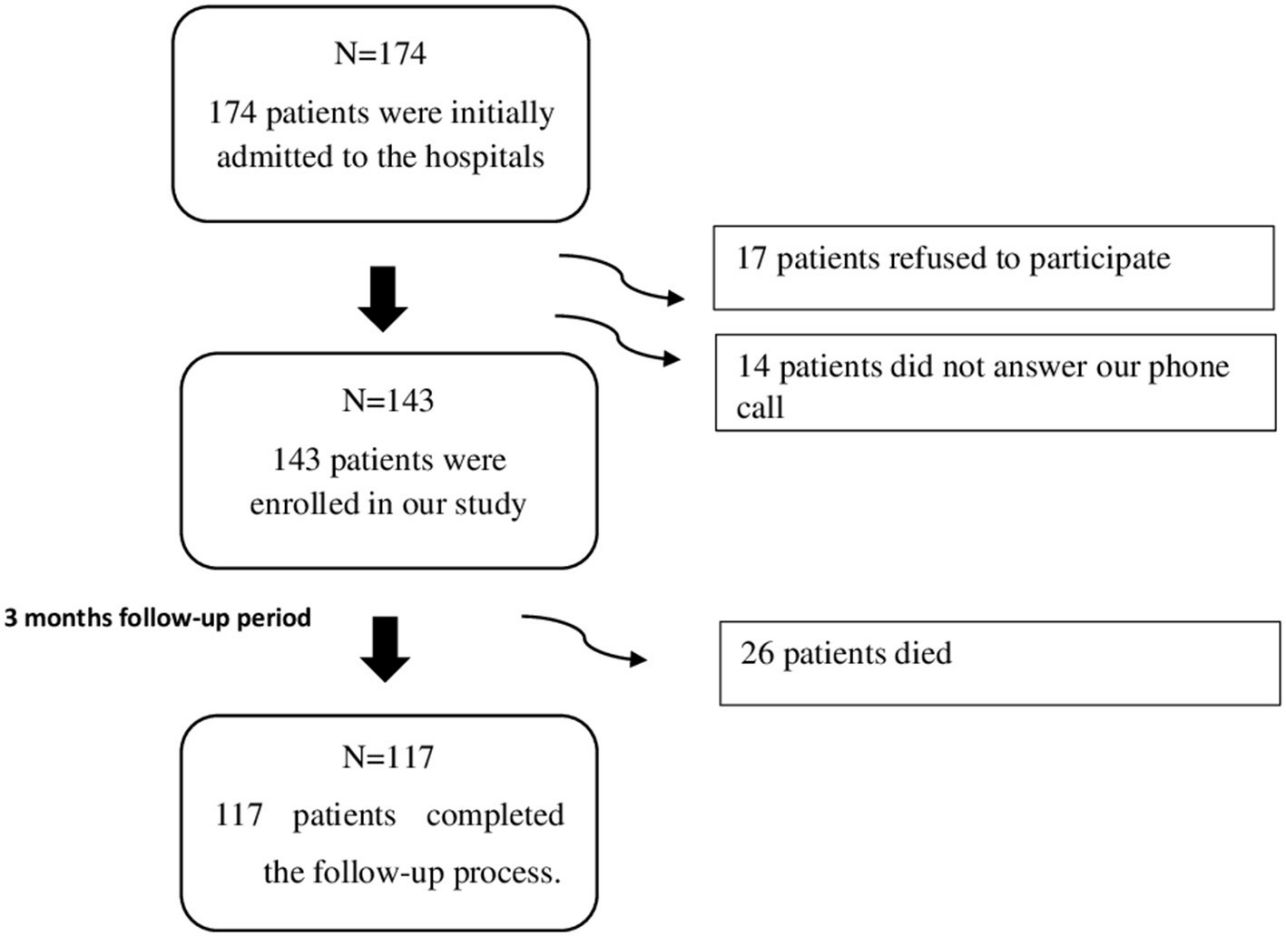
Flow chart outlining recruitment and drop out during the 3 months follow-up period.

Among the 174 patients admitted to the hospitals between February and June 2018, 143 patients were enrolled in our study, where 17 patients refused to participate, and 14 did not answer our phone call. The mean length of stay in the hospital of the patients was 9.78 (SD = 8.52) days. The patients recruited in our study were followed up for a 3 months' duration, through which 26 patients died, and the remaining 117 patients completed the follow-up process as shown in Figure 1.

### Baseline Characteristics of the Study Participants

Table 1 depicts the socio-demographic characteristics of the study sample. Of the total, 60.7% were males. The mean age was 72.46 (SD = 12.44) ranging from 33 to 95 and 60.7% of our sample were aged more than 70 years old, which confirm that stroke mainly occur in the elders. The majority (78.6%) were married. Concerning the education level, about one third (32.5%) did not receive any formal education and 49.6% had <12 years of education. Concerning the work status (44.4%) were unemployed and 22.2% were retired. Regarding current lifestyle, 63.6% of participants were smokers and 62.4% did not practice any physical activity. The majority of our sample (83.8%) had social security or insurance.

**Table 1:** Baseline characteristics of the study group.

	**All (*****N*** **= 117)**	
**Characteristics**	***N***	**(%)**
**Gender**		
Male	71	60.7
Female	46	39.3
**Age group (year)**		
33–50	5	4.3
50–70	41	35
>70	71	60.7
**Marital status**		
Married	92	78.6
Others^*^	25	21.4
**Education level**		
No formal education	38	32.5
<12 years	58	49.6
>12 years	21	17.9
**Employment**		
Employed	39	33.3
Unemployed	52	44.4
Retired	26	22.2
**Smoking status**		
No/occasionally	43	36.4
Yes	75	63.6
**Physical activity**		
No	73	62.4
Yes	44	37.6
**Sedentary lifestyle (Hours per day)**		
1–6	34	30.4
7–11	39	34.8
≥12	39	34.8
**Health Insurance**		
No	19	16.2
Yes	98	83.8

*N, frequency; %, percentage*.

* *Others stands for Single, divorced, or widowed*.

### Clinical Features of the Stroke Among the Patients

Concerning the type of stroke distributed among the patients, 112 of the 117 patients included (95.7%) were diagnosed with ischemic stroke, and 5 (4.3%) were diagnosed with intracerebral hemorrhage as shown in Table 2. Regarding the different locations of stroke among the patients, 42 patients (35.9%) had their stroke in their left hemisphere, 59 patients (50.4%) in the right hemisphere, 8 patients (6.8%) in their bilateral hemisphere, 7 patients (6%) in their cerebellum, and one patient in his brain stem (0.9%). When checking on their medical conditions, 80 patients (68.4%) have hypertension, 24 patients (20.5%) suffer from atrial fibrillation, and 50 patients (42.7%) suffer from dyslipidemia. Three months following stroke, 81 (69.2%, with a 95% CI of 60–77.4%), suffered from cognitive impairment, 110 (94%, with a 95% CI of 88.1–97.6%) from fatigue, and 29 (24.8%, with a 95% CI of 17.3–33.6%) from neuropathic pain (Table 2).

**Table 2:** Clinical features of the stroke and cognitive and psychological status in the study group.

	**All (*****N*** **= 117)**	
	***N***	**(%)**
**Type of Stroke**		
Ischemic Stroke	112	95.7
Intracerebral Hemorrhage	5	4.3
**Location of Stroke**		
Left hemisphere	42	35.9
Right hemisphere	59	50.4
Bilateral hemisphere	8	6.8
Cerebellum	7	6
Brain stem	1	0.9
**Hypertension (HTN)**		
No	37	31.6
Yes	80	68.4
**Atrial Fibrillation (AF)**		
No	93	79.5
Yes	24	20.5
**Dyslipidemia (DL)**		
No	67	57.3
Yes	50	42.7
**Cognitive Impairment (MMSE)**		
No	36	30.8
Yes	81	69.2
**Fatigue (FSS)**		
No	7	6
Yes	110	94
**Neuropathic Pain (DN4)**		
No	88	75.2
Yes	29	24.8

### Neurological Status of the Study Group at 3 Months After Stroke

Regarding the Neurological (NHISS) status of our sample size, the results showed that the majority of our patients were alert *n* = 105(89.7%), answer LOC Questions both correctly *n* = 95(81.2%), obey LOC commands both correctly *n* = 96(82.1%), have normal gaze *n* = 80(68.4%), have no visual loss *n* = 92(78.6%), have normal symmetrical movements of facial palsy *n* = 63(53.8%), have no drift; limb holds 90° (or 45°) for full 10 s of motor of left arm *n* = 76(65%), have no drift; limb holds 90° (or 45°) for full 10 s of motor of right arm *n* = 62(53%), have no drift; leg holds 30° position for full 5 s of motor of left leg *n* = 79(67.5%), have no drift; leg holds 30° position for full 5 s of motor of right leg *n* = 74(63.2%), have absent limb ataxia *n* = 54(46.2%), have normal; no sensory loss *n* = 95(81.2%), have mild to moderate aphasia *n* = 39(33.3%) and severe aphasia *n* = 39(33.3%), have normal dysarthria *n* = 43(36.8%), and severe dysarthria *n* = 43(36.8%), and have normal, no bending after 5 s of distal motricity *n* = 64(54.7%) (Table 3).

**Table 3:** Neurological status of the study group at 3 months after stroke.

	**All (*****N*** **= 117)**	
	***N***	**(%)**
**National Institute of Health Stroke Scale (NIHSS)**		
**Level of consciousness**		
Alert	105	89.7
Not alert, but arousable with minimal stimulation	6	5.1
Not alert, requires repeated stimulation to attend	6	5.1
Coma	0	0
**LOC Questions**		
Answers both correctly	95	81.2
Answers one correctly	6	5.1
Both incorrect	16	13.7
**LOC commands**		
Obeys both correctly	96	82.1
Obeys one correctly	5	4.3
Both incorrect	16	13.7
**Best gaze**		
Normal	80	68.4
Partial gaze palsy; gaze is abnormal in one or both eyes, but forced deviation or total gaze paresis is not present	34	29.1
Forced deviation, or total gaze paresis not overcome by the oculocephalic maneuver	3	2.6
**Visual fields**		
No visual loss	92	78.6
Partial hemianopsia	21	17.9
Complete hemianopsia	4	3.4
Bilateral hemianopsia	0	0
**Facial palsy**		
Normal symmetrical movements	63	53.8
Minor paralysis (flattened nasolabial fold, asymmetry on smiling)	31	26.5
Partial paralysis (total or near-total paralysis of lower face)	23	19.7
Complete paralysis of one or both sides (absence of facial movement in the upper and lower face)	0	0
**Motor of left arm**		
No drift; limb holds 90° (or 45°) for full 10 s	76	65
Drift; limb holds 90° (or 45°), but drifts down before full 10 s; does not hit bed or other support	14	12
Some effort against gravity; limb cannot get to or maintain (if cued) 90° (or 45°), drifts down to bed, but has some effort against gravity	15	12.8
No effort against gravity; limb falls	4	3.4
No movement	8	6.8
Amputation or joint fusion	0	0
**Motor of right arm**		
No drift; limb holds 90° (or 45°) for full 10 s	62	53
Drift; limb holds 90° (or 45°), but drifts down before full 10 s; does not hit bed or other support	27	23.1
Some effort against gravity; limb cannot get to or maintain (if cued) 90° (or 45°), drifts down to bed, but has some effort against gravity	13	11.1
No effort against gravity; limb falls	5	4.3
No movement	10	8.5
Amputation or joint fusion	0	0
**Motor of left leg**		
No drift; leg holds 30° position for full 5 s	79	67.5
Drift; leg falls by the end of the 5-s period but does not hit bed	12	10.3
Some effort against gravity; leg falls to bed by 5 s, but has some effort against gravity	12	10.3
No effort against gravity, leg falls to bed immediately	7	6
No movement	7	6
Amputation, joint fusion	0	0
**Motor of right leg**		
No drift; leg holds 30° position for full 5 s	74	63.2
Drift; leg falls by the end of the 5-s period but does not hit bed	19	16.2
Some effort against gravity; leg falls to bed by 5 s, but has some effort against gravity	8	6.8
No effort against gravity, leg falls to bed immediately	7	6
No movement	9	7.7
Amputation, joint fusion	0	0
**Limb ataxia**		
Absent	54	46.2
Present in one limb	26	22.2
Present in two limbs	37	31.6
Amputation or joint fusion	0	0
**Sensory**		
Normal; no sensory loss	95	81.2
Mild to moderate sensory loss; patient feels pinprick is less sharp or is dull on the affected side or there is a loss of superficial pain with pinprick but patient is aware he/she is being touched	19	16.2
Severe to total sensory loss; patient is not aware of being touched in the face, arm, and leg	3	2.6
**Language**		
Normal, no aphasia	36	30.8
Mild to moderate aphasia; some obvious loss of fluency or facility of comprehension, without significant limitation on ideas expressed or form of expression; reduction of speech and/or comprehension, however, makes conversation about provided material difficult or impossible.	39	33.3
Severe aphasia; all communication is through fragmentary expression; great need for inference, questioning, and guessing by the listener	39	33.3
Mute; global aphasia; no usable speech or auditory comprehension	3	2.6
**Dysarthria**		
Normal	43	36.8
Mild to moderate; patient slurs at least some words and, at worst, can be understood with some difficulty	30	25.6
Severe; patient's speech is so slurred as to be unintelligible in the absence of or out of proportion to any dysphasia, or is mute/anarthric	43	36.8
Intubated or other physical barrier	1	0.9
**Extinction and inattention**		
No abnormality	69	59
Visual, tactile, auditory, spatial, or personal inattention or extinction to bilateral simultaneous stimulation in one of the sensory modalities	44	37.6
Profound hemi-inattention or hemi-inattention to more than one modality; does not recognize own hand or orients to only one side of space	4	3.4
**Distal motricity**		
Normal, no bending after 5 s	64	54.7
Maintain an extension after 5 s, but it's incomplete	23	19.7
No voluntary extension after 5 s	30	25.6
Amputation or joint fusion	0	0

### Frequency of Post-Stroke Psychological Complications Among Patients

**Figure 2:**
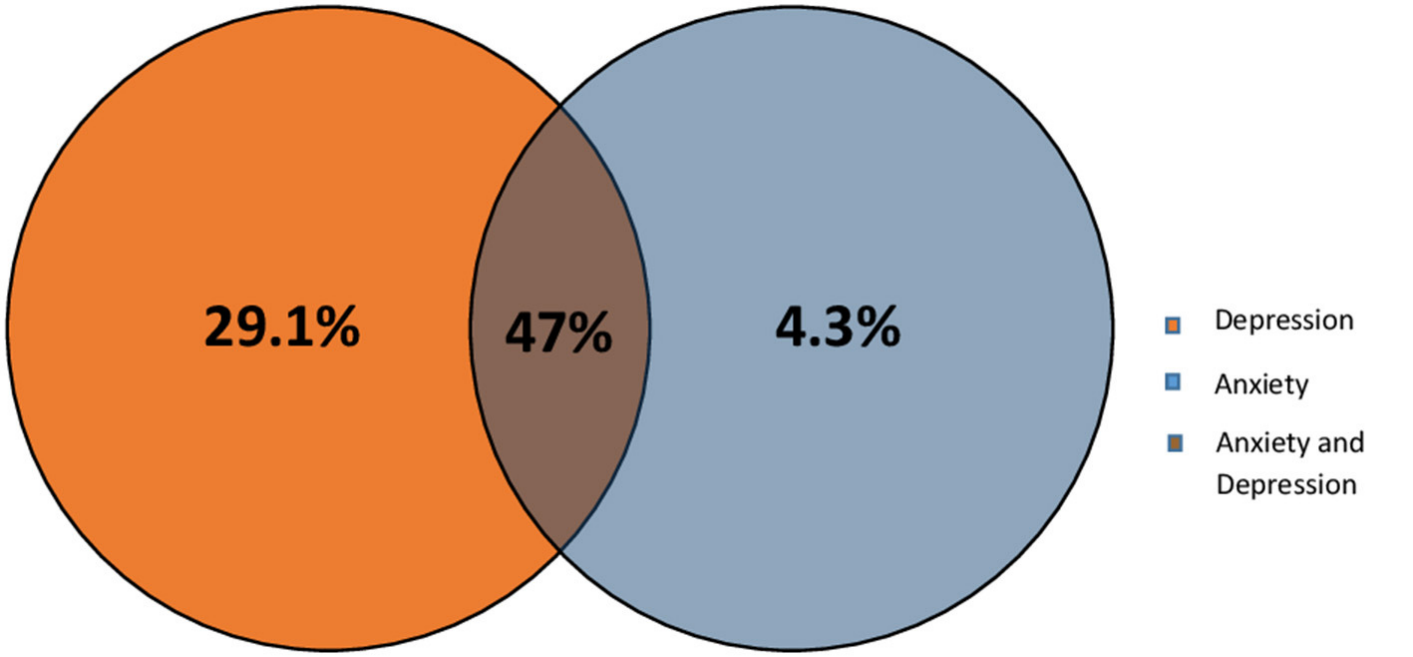
Post stroke psychological complications among patients.

Previous studies have shown that most of the stroke patients suffer from depression and/or anxiety (Barker-Collo, 2007). Therefore, we wanted to check the level of each of these conditions within our Lebanese patients using HADS scale. Results shows that at the end of the first 3 months, 34 (29.1%, 95% CI 21–38.2%) of the patients experienced only depression, and 4.3%, with a 95% CI of 1.4–9.7% experienced only anxiety, however 55 (47%, 95% CI 37.7–56.5%) of the patients experienced both anxiety and depression (Based on HADS scale; score ≥8 for depression and anxiety subscales) as shown in Figure 2.

### Correlates of Depression 3 Months After Stroke Among Lebanese Patients

Depression, a frequent neuropsychiatric disorder after stroke, is commonly encountered by stroke survivors. In addition, its prevalence and severity are affected with several factors. The results of the bivariate analysis of the HADS depression scores with variables related to post stroke depression (based on literature) are presented in Table 4. Physical activity, Mini-Mental State Examination (MMSE), Hospital Anxiety and Depression Scale (HADS-A), Douleur Neuropathique 4 **(**DN4), Fatigue Severity Scale (FSS), and National Institutes of Health Stroke Scale (NIHSS) were associated with depression in bivariate analysis (*P*-value <0.05). However, logistic regression analysis revealed that HADS-A, DN4, NHISS, and physical activity were significantly associated with depression (Table 5). The odds of depression in patients that experienced post stroke anxiety was 4.814 times the odds of depression in non-anxious patients (OR = 4.814, *p*-value = 0.017), and the odds of depression in patients that experienced post stroke pain was 6.868 times more than that in patients that didn't experienced pain (OR = 6.868, *p*-value = 0.002), while physical activity was found to be inversely correlated with depression (OR = 0.261; *p*-value = 0.029). Moreover, NHISS increases the odds of depression by 1.274 times.

**Table 4:** Bivariate analysis of depression 3 months after stroke in the study group.

**Variable**	**No Depression**	**Depression**	**Unadjusted OR**	***P*-value**
	***n* (%)**	***n* (%)**	**(95% CI)**	
**Gender**				0.65
Male	18 (25.4)	53 (74.6)	1.0	
Female	10 (21.7)	36 (78.3)	1.2 (0.5–3)	
**Physical activity**				**<0.0001**
No	8 (11.0)	65 (89.0)	1.0	
Yes	20 (45.5)	24 (54.5)	0.15 (0.06–0.38)	
**Employment status**				0.468
Employed	11 (21.2)	41 (78.8)	1.0	
Unemployed	12 (30.8)	27 (69.2)	0.60 (0.23–1.56)	
Retired	5 (19.2)	21 (80.8)	1.13 (0.35–3.67)	
**Educational Level**				0.169
No formal education	6 (15.8)	32 (84.2)	1	
<12 years	14 (24.1)	44 (75.9)	0.589 (0.2–1.7)	
>12 years	8 (38.1)	13 (61.9)	0.305 (0.09–1.05)	
**MMSE**				**0.011**
≤ 23	149 (38.9)	22 (61.1)	1.0	
>23	14 (17.3)	67 (82.7)	3.04 (1.26–7.37)	
**HADS-A**				**<0.0001**
≤ 8	23 (40.4)	34 (59.6)	1	
>8	5 (8.3)	55 (91.7)	7.44 (2.59–21.42)	
**DN4**				**<0.0001**
≤ 4	22 (47.8)	24 (52.2)	1.0	
>4	6 (8.3)	64 (91.4)	9.8 (3.54–27.04)	
**FSS**				**<0.0001**
≤ 4	6 (85.7)	1 (14.3)	1.0	
>4	22 (20)	88 (80)	24 (2.75–209.8)	
**NIHSS (mean.SD**)	4.6 (2.42)	12.79 (8.85)	1.274 (1.12–1.45)	**<0.001**

*n, frequency; %, percentage; OR, odds Ratio; CI, Confidence interval; MMSE, Mini-Mental State Examination; DN4, Douleur Neuropathique 4; HADS-A, Hospital anxiety and depression scale- Anxiety, subscale; FSS, Fatigue Severity, NHISS Scale. P-value <0.05 is considered significant*.

**Table 5:** Adjusted odds ratios with their 95% confidence intervals from the logistic regression of depression at 3 months after stroke in the study group.

**Variable**	**Adjusted OR**	**95% CI**	***P*-value**
**Physical activity**			**0.029**
No	1	1	
Yes	0.261	0.079–0.87	
**Educational level**			0.885
No formal education	1	1	
<12 years	0.995	0.24–4.135	
>12 years	0.71	0.125–4.02	
**MMSE**			0.812
≤ 23	1	1	
>23	1.177	0.307–4.51	
**HADS-A**			**0.017**
No	1	1	
Yes	4.814	1.331–17.405	
**DN4**			**0.002**
≤ 4	1	1	
>4	6.868	2.013–23.438	
**FSS**			0.573
≤ 4	1	1	
>4	2.052	0.169–24.986	
**NIHSS**	1.274	1.12–1.45	**<0.001**

*OR, odds Ratio; CI, Confidence interval; MMSE, Mini-Mental State Examination; HADS-A, Hospital anxiety and depression scale- Anxiety, subscale; DN4, Douleur Neuropathique 4; FSS, Fatigue Severity Scale, NHISS scale. P-value <0.05 is considered significant*.

### Correlates of Anxiety 3 Months After Stroke Among Lebanese Patients

Anxiety is one of the prominent consequences of stroke. Several factors play important roles in affecting its existence and severity post stroke. The results of the bivariate analysis of the HADS anxiety scores with variables related to post stroke depression (based on literature) are presented in Table 6. Educational level, physical activity, sedentary duration, AF, DL, neuropathic pain, and NHISS scale were associated with anxiety in bivariate analysis (*P*-value <0.05). Additionally, a multivariate logistic regression analysis was performed in order to adjust the independent effects of the variables. Non-significant Hosmer and Lomeshow Test (0.103) were found, and Nagelkerke R square: *R*^2^ = 0.391: 39.1% of variance of the dependent variables was explained by the independent variables. The results of the multivariate logistic regression analysis as shown in Table 7 revealed that sedentary life style, neuropathic pain (DN4 ≥ 4), and NHISS scale during the first 3 months post-stroke were significantly associated with anxiety. Anxiety is more likely to occur in case of extended sedentary duration. Patients with longer sedentary duration (between 7 and 11 h per day) were 4.123 more likely to develop post stroke anxiety compared to those with shorter sedentary duration (1–6 h per day). Moreover, patients with sedentary duration longer than 12 h were 8.457 times more likely to develop post stroke anxiety compared to their counterparts with sedentary duration of 1–6 h per day. Furthermore, patients with neuropathic pain are 3.858 times more likely to have anxiety compared to patients without neuropathic pain (ORa = 3.858, *p*-value = 0.019). Moreover, NHISS increases the odds of anxiety by 1.198 times.

**Table 6:** Bivariate analysis of anxiety 3 months post stroke in the study group.

**Variable**	**No Anxiety**	**Anxiety**	**Unadjusted OR**	***P-*value**
	***n*(%)**	***n*(%)**	**(95% CI)**	
**Gender**				0.877
Male	35 (49.3%)	36 (50.7%)	1	
Female	22 (47.8%)	24 (52.2%)	1.061 (0.51–2.23)	
**Educational Level**				**0.025**
No formal education	12 (31.6%)	26 (68.4%)	1	
<12 years	31 (53.4%)	27 (46.6%)	0.402 (0.17–0.95)	
>12 years	14 (66.7%)	7 (33.3%)	0.231 (0.07–0.72)	
**Physical Activity**				**0.004**
No	28 (38.4%)	45 (61.6%)	1	
Yes	29 (65.9%)	15 (34.1%)	0.322 (0.15–0.703)	
**Sedentary Duration**				**<0.001**
1–6 h	26 (76.5%)	8 (23.5%)	1	
7–11 h	18 (46.2%)	21 (53.8%)	3.792 (1.38–10.43)	
>12 h	8 (20.5%)	31 (79.5%)	12.594 (4.15–38.21)	
**Hypertension (HTN)**				0.42
No	16 (43.2%)	21 (56.8%)	1	
Yes	41 (51.2%)	39 (48.8%)	0.725 (0.33–1.59)	
**Atrial Fibrillation (AF)**				0.091
No	49 (52.7%)	44(47.3%)	1	
Yes	8 (33.3%)	16 (66.7%)	2.227 (0.87–5.71)	
**Dyslipidemia (DL)**				0.173
No	29 (43.3%)	38 (56.7%)	1	
Yes	28 (56%)	22 (44%)	0.6 (0.29–1.25)	
**DN4**				**0.002**
≤ 4	50 (56.8%)	38 (43.2%)	1	
>4	7 (24.1%)	22 (75.9%)	4.135 (1.6–10.69)	
**NIHSS (mean, SD)**	6.15 (4.83)	15.25 (9.03)	1.198 (1.12–1.29)	**<0.001**

*n, frequency; %, percentage; OR, odds Ratio; CI, Confidence interval; HTN, Hypertension; DN4, Douleur Neuropathique 4, NHISS, P-value <0.05 is considered significant*.

**Table 7:** Adjusted odds ratios with their 95% confidence intervals from the logistic regression of anxiety at 3 months after stroke in the study group.

**Variable**	**Adjusted OR**	**95% CI**	***P*-value**
**Educational Level**			0.62
No formal education	1	1	
<12 years	0.624	0.214–1.823	
>12 years	0.968	0.225–4.16	
**Physical activity**			0.228
No	1	1	
Yes	0.501	0.163–1.541	
**Sedentary Duration**			**0.01**
1–6 h	1	1	
7–11 h	4.123	1.23–13.823	
>12 h	8.457	2.082–34.354	
**AF**			0.081
No	1	1	
Yes	2.897	0.879–9.551	
**DL**			0.073
No	1	1	
Yes	0.408	0.153–1.085	
**DN4**			**0.019**
≤ 4	1	1	
>4	3.858	1.243–11.974	
**NIHSS**	1.198	1.12–1.29	**<0.001**

*OR, odds Ratio; CI, Confidence interval; AF, Atrial Fibrillation; DL, Dyslipidemia; DN4, Douleur Neuropathique 4, NHISS. P-value <0.05 is considered significant*.

## Discussion

Anxiety and depression are common and major consequences following stroke. About 30% of stroke survivors experience depression, while anxiety prevalence is around 20–25% (Fang et al., 2017). This study aimed to investigate the psychological consequences of a stroke among Lebanese survivors 3 months following the event and to identify their correlates. At the end of the first 3 months, 89 (76.1%) of the patients experienced depression and 51.3% experienced anxiety. Anxiety, neuropathic pain, physical activity, and NHISS scale were correlated with depression. However, sedentary life style, neuropathic pain, and NHISS scale during the first 3 months post-stroke were significantly associated with anxiety.

Previous studies indicated that post stroke depression is the commonest mental disorder in survivors. Our study indicated a high level of depression (76.1%) recorded 3 months following stroke compared to other studies conducted in western countries which indicated a prevalence of 45.6% (Barker-Collo, 2007), and 39% (Hosking et al., 2000). A possible explanation for this difference could be the general poor feature of life following stroke, especially among the Lebanese population, which are deprived of good care and rehabilitation facilities, physical and social restrictions following stroke, and lack of additional training for healthcare professionals on the symptoms of depression (Kaadan and Larson, 2017). Another possible explanation could be the usage of different methods of assessment (Hackett et al., 2005).

Depression has negative effects on functional recovery, survival, cognitive and social functions, where it is usually associated with several factors (Bolla-Wilson et al., 1989; Kauhanen et al., 1999). We further investigated the elements affecting depression. Our results revealed that physical activity acts as a protective factor against depression 3 months following stroke. It improves the patient's self-esteem, increases their sense of comfort, and happiness due to elevated serotonin and noradrenergic synthesis following exercise, which consequently diminish depression levels (Anderson and Shivakumar, 2013). Similarly, these results have been reported by two recent systematic reviews, thus spotting the light on the importance of intervention to increase physical activity (Pengpid and Peltzer, 2019). Neuropathic pain revealed a significant association with depression as well. These findings are similar to a previous review of 40 studies indicating that pain is a causative factor for depression (Woo, 2010). This could be clarified by the fact that pain could lead to impaired functioning, and thus causes social isolation, which in turn upsurges depression levels (Mushtaq et al., 2014). Furthermore, our study indicated a correlation between anxiety and depression, which was consistent with previous studies (Fang et al., 2017). Researchers have tested hypotheses of shared genetic etiologies as a potential basis of this relationship (Hettema, 2008). Moreover, our results showed that there is a significant association between NHISS scale and depression 3 months after stroke (*p*-value <0.001), which was consistent with the study of Ilut et al. (2017) that showed that higher NIHSS scores were associated with severe depression (Ilut et al., 2017).

Post-stroke anxiety is another common neuropsychiatric consequence prevalent after stroke. About one-quarter of stroke survivors experience post-stroke anxiety that affects their rehabilitation due to its negative impact on quality of life (QOL) (Li et al., 2019). It is associated with immobility, fatigue and poor self-control (Fang et al., 2017). Many studies indicated the persistence and mutuality of post-stroke anxiety (Cumming et al., 2016). Through our investigation, the frequency of anxiety (51.7%) was not consistent with other studies that demonstrated lower frequency after 3 months of stroke, with an estimated prevalence ranging between 18 and 38% (Barker-Collo, 2007; Ayerbe et al., 2014; Vansimaeys et al., 2017). The high level of anxiety in Lebanon could be related to the lack of rehabilitative services following stroke, the lack of social and psychological support for the survivor, and the high burden of physical disability on them.

The severity of anxiety syndrome after a stroke is usually linked to a general poor feature of life following stroke (Rafsten et al., 2018). Our study indicated a relationship between anxiety and sedentary duration (*p*-value = 0.01), which was confirmed by a previous systematic review (Teychenne et al., 2015). Long sedentary behavior seems to be related to anxiety at the biological and the psychosocial levels. Biologically, sedentary behavior might lead to central nervous system arousal, sleep disturbances, or poor metabolic health. Alternatively, prolonged sedentary behaviors may be linked to social withdrawal theory. For example, prolonged television viewing, may lead to social solitude and withdrawing from interpersonal relationships, which has been linked to increased feelings of social anxiety (Teychenne et al., 2015). Furthermore, our study confirmed a direct relation between anxiety and pain. This is explained by the fact that stroke survivors who suffer from pain usually pay increased attention toward threat, which increases their anxiety levels toward any physical work (de Heer et al., 2014). Moreover, our results showed that there is a significant association between NHISS scale and anxiety three months after stroke (*p*-value <0.001), which was consistent with the study of Lee et al. (2019) that revealed that post stroke anxiety was significantly associated with a higher NIHSS score.

To the best of our knowledge, this is the first prospective study examining post-stroke consequences done among first-ever stroke survivors in Lebanon. The data is collected through a face-to-face interview and completed by the investigator himself, and thus, the degree of bias usually resulting from self-completed questionnaires due to a misunderstanding of the questions was declined. However, regarding the limitations, our study had a small sample size, and the risk of selection bias due to the convenience sampling method used to select patients might have restricted the capacity to generalize our findings to all the Lebanese stroke survivors. It should be mentioned we have used the Arabic validated version of the HADS (Al Aseri et al., 2015), Dn4 (Terkawi et al., 2017), FSS (Al-Sobayel et al., 2016), NHISS (Hussein et al., 2015) which is considered a strength with regard to information bias. Nevertheless, additional studies are required to confirm the current study results.

## Conclusion

Neuropsychological disorders are generally debilitating for patients and they impose a socioeconomic burden on these patients and their caregivers. The current study provides pioneer information about the consequences of stroke 3 months following the event, mainly neuropsychiatric events affecting functional recovery. Special care should be provided for patients presenting risk factors for anxiety and depression, such as extended sedentary durations, low physical activity, and high pain rates. These psychiatric events represent a major complication in front of the recovery mechanism where they could lead to less compliance to medications, higher fatigue levels, and therefore poorer outcomes. The high frequency of neuropsychiatric complications provided by this study in Lebanon will give much more attention on post-stroke complications and highlight the need for further investigations into the pathogenesis, prevention, and treatment. Thus, necessary protocols should be established for early detection and treatment, and an action plan should be conducted to prevent, manage stroke during the acute phase, evaluate its outcome, and organize the rehabilitation services concerning the consequences following stroke. The rehabilitation process needs to be considered in its broadest context, and include an equal focus on the social, psychological and physical aspects. Additionally, the inclusion of community-based rehabilitation workers is critical to the success of any comprehensive, cost-effective rehabilitation paradigm.

## Data Availability Statement

The raw data supporting the conclusions of this article will be made available by the authors, without undue reservation.

## Ethics Statement

The studies involving human participants were reviewed and approved by Neuroscience Research Committee, at the NRC, Lebanon. The patients/participants provided their written informed consent to participate in this study.

## Author Contributions

WK, MT, and CB undertook the data collection process. WK and MT undertook data management, statistical analysis, and drafted the manuscript. LA-A critically revised the data analysis part. NS, PS, and LA-A contributed to the final draft of the manuscript and gave final approval for submission. All authors contributed to the article and approved the submitted version.

## Conflict of Interest

The authors declare that the research was conducted in the absence of any commercial or financial relationships that could be construed as a potential conflict of interest.

## Abbreviations

WHO: World Health Organization
GBD: Global Burden of Diseases
VCI: Vascular Cognitive Impairment
VaD: Vascular Dementia
PSD: Post-stroke depression
DSM: Diagnostic and Statistical Manual
ICD-10: International Classification of Diseases 10
TIA: Transient Ischemic Attack
IRB: Institutional Review Board
HTN: Hypertension
AF: Atrial Fibrillation
DL: Dyslipidemia
MMSE: Mini-Mental State Examination
HADS: Hospital Anxiety and Depression Scale
FSS: Fatigue Severity Scale
DN4: Douleur Neuropathique 4
SPSS: Statistical Package for Social Sciences
QOL: Quality of Life.
